# Further Mechanisms and Locations in Which Antisporozoite Antibodies Neutralize Malaria Sporozoites

**DOI:** 10.1128/mBio.01588-19

**Published:** 2019-09-10

**Authors:** Jerome P. Vanderberg

**Affiliations:** a New York University School of Medicine, New York, New York, USA; NIAID/NIH; University of California Los Angeles

**Keywords:** antibodies, malaria, preerythrocytic, skin, sporozoites, vaccine, immune complexes, antisporozoite antibodies, circumsporozoite protein, immune complexes, *Plasmodium*, sporozoite

## LETTER

Having worked in this area for many years, I read with much interest the recent article “Antibody-Mediated Protection against *Plasmodium* Sporozoites Begins at the Dermal Inoculation Site” by Flores-Garcia et al. (1). It is now universally recognized, as described by the authors, that mosquitoes inject most of their sporozoites into extravascular skin tissue rather than directly into blood vessels (2, 3). From there, they migrate through the dermis to enter blood vessels (3) and are carried to the liver, where they invade hepatocytes to continue their development. The authors further note that sporozoites spend much of their extracellular time at the skin inoculation site, thus rendering them vulnerable to antibody-mediated destruction at this site. Such antibody-mediated immobilization or destruction of sporozoites has been shown both *in vitro* and *in vivo* (3–5). I am gratified that the authors have corroborated these prior findings made by us and several other authors and have expanded them with new data.

The authors concluded that antibodies targeting the migratory sporozoites exert a large proportion of their protective effect at the inoculation site but that the mechanisms by and location in which they neutralize parasites have not been fully elucidated (1). I would like, however, to call attention to the evidence we presented in our 2009 publication (5) in which we elucidated an entirely new and complementary way in which sporozoites can be neutralized by host antibodies.

It is known that living sporozoites release large quantities of soluble circumsporozoite protein (CSP) into their environment both *in vitro* and *in vivo* and that soluble CSP is found within the saliva of malaria-infected mosquitoes (6, 7). Thus, infected mosquitoes introduce into the skin of immunized hosts not only CSP-covered sporozoites but also soluble CSP, both of which encounter and interact with homologous anti-CSP antibodies *in situ* within avascular tissue of the host dermis. We presented evidence that many of the sporozoites are trapped there within apparent immune complexes, as determined by confocal microscopy and specific staining with fluorescein isothiocyanate (FITC)-conjugated protein A and A/C. Thus, sporozoites were not only immobilized by CS antibodies as has previously been shown but were additionally entrapped by being encased within these immune complexes. I respectfully suggest, in disagreement with the conclusions of the authors (1), that the mechanisms by and location in which antisporozoite antibodies neutralize parasites have indeed been further elucidated, as demonstrated in our 2009 paper (5).

## References

[1] Flores-Garcia Y, Nasir G, Hopp CS, Munoz C, Balaban AE, Zavala F, Sinnis P 2018 Antibody-mediated protection against *Plasmodium* sporozoites begins at the dermal inoculation site. mBio 9:e02194-18. doi:10.1128/mBio.02194-18. 30459199 PMC6247089

[2] Sidjanski S, Vanderberg JP 1997 Delayed migration of *Plasmodium* sporozoites from the mosquito bite site to the blood. Am J Trop Med Hyg 57:426–429. doi:10.4269/ajtmh.1997.57.426. 9347958

[3] Vanderberg JP, Frevert U 2004 Intravital microscopy demonstrating antibody-mediated immobilisation of *Plasmodium berghei* sporozoites injected into skin by mosquitoes. Int J Parasitol 34:991–996. doi:10.1016/j.ijpara.2004.05.005. 15313126

[4] Stewart MJ, Nawrot R, Schulman S, Vanderberg JP 1986 *Plasmodium berghei* sporozoite invasion is blocked in vitro by sporozoite-immobilizing antibodies. Infect Immun 51:859–864. 3512436 10.1128/iai.51.3.859-864.1986 PMC260977

[5] Kebaier C, Voza T, Vanderberg JP 2009 Kinetics of mosquito-injected *Plasmodium* sporozoites in mice: fewer sporozoites are injected into sporozoite-immunized mice. PLoS Pathog 5:e1000399. doi:10.1371/journal.ppat.1000399. 19390607 PMC2667259

[6] Stewart MJ, Vanderberg JP 1991 Malaria sporozoites release circumsporozoite protein from their apical end and translocate it along their surface. J Protozool 38:411–421. doi:10.1111/j.1550-7408.1991.tb01379.x. 1787427

[7] Beier JC, Vaughan JA, Madani A, Noden BH 1992 *Plasmodium falciparum*: release of circumsporozoite protein by sporozoites in the mosquito vector. Exp Parasitol 75:248–256. doi:10.1016/0014-4894(92)90185-D. 1516673

